# Occupational exposure to pesticides and health symptoms among farmers in Palestine

**DOI:** 10.4314/ahs.v24i4.46

**Published:** 2024-12

**Authors:** Iyad Ali

**Affiliations:** Department of Medical Sciences, Faculty of Medicine and Health Sciences, An-Najah National University, Nablus, Palestine

**Keywords:** Pesticides, farmers, toxicity, symptoms, Palestine

## Abstract

**Background:**

This study aimed to examine the usage patterns of commonly used pesticides and categorize the health problems associated with long-term exposure.

**Methods:**

Data for this cross-sectional study was collected between February 2020 and October 2022. Participants from various agricultural regions were recruited through social media surveys.

**Results:**

This study included 1105 farmers from diverse agricultural areas, and found that the participants' level of education was a significant factor in determining the number of reported symptoms. Significant correlations were also found between the number of symptoms and the frequency and duration of pesticide spraying, as well as the use of personal protective equipment. A small percentage of participants reported no symptoms and were excluded from the analysis. Negative associations were observed between the number of symptoms and age group, high school education, frequency of pesticide use per week, involvement in other work, and source of information. Positive associations were found between the number of symptoms and spraying period and perception of pesticide toxicity.

**Conclusion:**

Farmers exposed to pesticides experienced various symptoms and illnesses, including cardiovascular, dermatological, neurological, and hematological symptoms. The number of reported symptoms decreased significantly with higher education levels and the use of personal protective measures.

## Introduction

Agricultural pesticides are crucial tools for farmers, used to counteract the negative impact of pests on crop growth and yield^1^. Formulated to protect crops from potential harm, these chemicals vary widely in mechanism, absorption, metabolism, elimination, and toxicity levels to humans. Pesticides with high acute toxicity, easily metabolized and eliminated, pose the greatest risk during short-term exposure. Conversely, those with lower acute toxicity but the potential for accumulation present a heightened risk during long-term exposure, even at low doses^2^.

Extended exposure to pesticides can lead to symptoms associated with cardiovascular diseases, neurological disorders, hematological abnormalities, and skin conditions^3^. Lack of awareness among farmers about the adverse impacts may lead to overestimation of the benefits, resulting in excessive and suboptimal use that can be detrimental to both individuals and the community^4^. While the exact number of pesticide poisonings is unknown, estimates indicate that India alone experiences 1-1.5 million cases per year, with approximately one-third resulting in fatalities^5^.

Farmers, during various stages of the crop preparation process, face frequent exposure to pesticides. The unregulated and uninformed use of these chemicals can directly impact farmers and nearby communities through pesticide drift and residues in food and water^6^. Studies in low- and middle-income countries reveal acute pesticide poisoning as a significant contributor to illness and mortality rates among farmworkers^7^. Additionally, long-term exposure is associated with chronic health effects, such as alterations in neurobehavioral function, respiratory problems, obesity, and diabetes^8^.

Globally, farmers invest around USD 40 billion annually in applying pesticides, with bio-pesticides accounting for just 2% of the entire crop-protection industry^9^. Farmers in highly developed nations typically anticipate a return of about five times the amount spent on pesticides^1^. Quick toxicity tests and tools provide valuable information about the symptoms of human risks and incidents of pesticide self-poisoning. This information can assist decision-making in fields such as pesticide management regulation, environmental evaluation, and emergency situations^10^.

Unsafe pesticide use poses significant risks to human health, emphasizing the importance of utilizing pesticides safely and following proper hygiene practices^11^. Despite the well-known harmful effects of pesticides, various studies have demonstrated their widespread use^12^, with farmers often neglecting necessary precautions to reduce the risk of pesticide poisoning^13^.

Understanding farmers' safety behaviors is crucial for promoting sustainable agriculture and enhancing intervention programs^14^. A survey involving 381 predominantly male farm workers revealed limited knowledge and adherence to safety procedures. Participants scored an average of 2.8 out of 8 for knowledge and 9.8 out of 15 for safety procedures, with a positive correlation observed between the two^15^. Al-Rujoub et al recommended enhancing occupational safety for farmers at the Palestinian level through the adoption of safety equipment and the improvement of preventive measures^16^. This study aims to evaluate how do the application practices of commonly used pesticides among farmers contribute to potential health issues resulting from long-term exposure, and what factors influence farmers' safety behaviors in the context of pesticide usage?

## Methods

An online survey was employed in this study to investigate the use of pesticides and associated health issues among Palestinian farmers. This study focused on Palestinian farmers who regularly use pesticides (individuals engage in the consistent application of chemical substances to control pests independently, without relying on external help) in their agricultural activities. The survey, conducted from February 2020 to October 2022, encompassed 1,105 farmers actively engaged in pesticide use across various Palestinian agricultural settings, with a commendable response rate of 78%. The sample size was determined using the Raosoft Sample Size Calculator, and participants were chosen through a random selection process.. The questionnaire was disseminated across various Palestinian social media pages, particularly those dedicated to agricultural matters, and it was completed online by farmers who willingly volunteered to take part in the study. The survey comprised four parts and 31 questions, which evaluated demographics, farming experience, pesticide application methods, knowledge of pesticide toxicity and safety precautions, and self-reported symptoms. The chi-square test was used to analyze associations between categorical variables, with a significance level of p<0.050. We utilized regression models as a statistical analysis technique to examine and interpret the data. In our analysis, Poisson regression models were employed to investigate the relationship between various independent variables and the dependent variable, which pertained to the count of reported symptoms. To establish the definitive adjusted model, we conducted a logistic regression analysis, incorporating all statistically significant analytical variables The Institutional Review Board at An-Najah National University, Nablus, Palestine, approved the study (IRB22/19), and participants were recruited through social media platforms. Responding to all questions was mandatory.

## Results

In this study, 1105 Palestinian farmers from different agricultural regions participated, resulting in a response rate of more than 97.5%, as demonstrated in Table 1. The majority of participants were male and mostly aged between 41 and 50 years old. Most of the farmers were married and smokers. Over one-third of them had more than 20 years of experience in agriculture, and around 45% were high school graduates. Approximately 43% of the farmers were involved in both open-land and greenhouse farming. Two-thirds of the farmers used pesticides once a week and sprayed them for an average of 10 to 30 minutes. During spraying, more than 75% of farmers wore facemasks, and 98% washed their hands afterward. While mixing pesticides, 78% of farmers wore gloves, and 55% wore eyeglasses. Six percent of farmers did not follow the instructions for preparing pesticides, but two-thirds wore special clothes. Around 6% of farmers may consume food or smoke while spraying pesticides. Nearly all farmers washed fruits and vegetables before eating them (97%), and 98% never reused pesticide containers. Only 35% of farmers consulted agriculture engineers, and only 5% of farmers kept pesticides in their homes. About 58% of participants believed that all pesticides are hazardous, and 50% of farmers used various pesticides in the same way.

**Table 1:** Population Characteristics and Relationship between the participants' characteristics and number of symptoms

Variable	Total n (%)	Number of symptoms			

		Mean ± SD	MD (Q1-Q3)	Min, Max	*p*-value
**Age**					
≤40	372(33.67%)	4.36 ± 2.7	4(2-6)	18,1	0.01^a^
>40	715(64.71%)	3.93 ± 2.54	3(2-5)	14,1	

**Gender**					
Female	103(9.32%)	3.98 ± 2.63	4(1.5-6)	10,1	0.67^a^
Male	978(88.51%)	4.09 ± 2.6	4(2-6)	18,1	

**Marital status**					
Married	931(84.25%)	4.05 ± 2.56	4(2-6)	18,1	0.44^a^
Unmarried	172(15.57%)	4.22 ± 2.76	4(2-6)	12,1	

**Smoking**					
No	789(71.4%)	4.16 ± 2.59	4(2-6)	18,1	0.09^a^
Yes	314(28.42%)	3.86 ± 2.61	3(2-5)	13,1	

**Education (high school)**					
Yes	868(78.55%)	3.97 ± 2.58	4(2-6)	18,1	0.01^a^
No	235(21.27%)	4.45 ± 2.61	4(3-6)	13,1	

**Years of working**					
<10 years	315(28.51%)	4.15 ± 2.62	4(2-5)	18,1	0.80^a^
≥10 years	887(71.31%)	4.05 ± 2.58	4(2-6)	14,1	

**Types of crops**					
Green house	95(8.6%)	3.55 ± 2.66	3(1-5)	13,1	0.01^a^
Open-land crops	531(48.05%)	3.94 ± 2.51	4(2-5)	18,1	
Both	477(43.17%)	4.33 ± 2.65	4(2-6)	14,1	

**Frequency of spraying**					
1/week	739(66.88%)	3.75 ± 2.46	3(2-5)	18,1	<0.001^a^
>1/week	(32.94%)	4.74 ± 2.73	4(3-6.25)	13,1	

**Spraying duration**					
<30	523(47.33%)	3.76 ± 2.53	3(2-5)	18,1	<0.001^a^
≥30	580(52.49%)	4.36 ± 2.62	4(2-6)	14,1	

**Wearing mask**					
No	250(22.62%)	3.61 ± 2.35	3(2-5)	13,11	<0.001^a^
Yes	853(77.19%)	4.21 ± 2.65	4(2-6)	8,1	

**Washing hands**					
No	17(1.54%)	3.35 ± 2.09	4(1-4)	7,1	0.25^a^
Yes	1086(98.28%)	4.09 ± 2.6	4(2-6)	18,1	

**Eating/smoking while spraying**					
No	1033(93.48%)	4.07 ± 2.58	4(2-6)	14,1	0.89^a^
Yes	70(6.33%)	4.11 ± 2.77	4(2.25-5)	18,1	

**Wearing gloves**					
No	861(77.92%)	4.21 ± 2.66	4(2-6)	18,1	<0.001^a^
Yes	242(21.9%)	3.6 ± 2.29	3(2-5)	12,1	

**Other work**					
No	733(66.33%)	4.31 ± 2.64	4(2-6)	18,1	<0.001^a^
Yes	370(33.48%)	3.61 ± 2.44	3(2-5)	13,1	

**Wearing eyeglasses**					
No	495(44.8%)	3.67 ± 2.3	3(2-5)	11,11	<0.001^a^
Yes	608(55.02%)	4.4 ± 2.77	4(2-6)	8,1	

**Washing vegetables**					
No	35(3.17%)	3.71 ± 2.14	3(2-5)	9,1	0.40^a^
Yes	1068(96.65%)	4.09 ± 2.61	4(2-6)	18,1	

**Special cloths**					
No	358(32.4%)	3.69 ± 2.3	3(2-5)	12,1	<0.001^a^
Yes	745(67.42%)	4.26 ± 2.71	4(2-6)	18,1	

**Reuse pesticide boxes**					
No	1078(97.56%)	4.07 ± 2.57	4(2-6)	14,1	0.58^a^
Yes	25(2.26%)	4.36 ± 3.51	4(2-5)	18,1	

**Adhere to guidelines**					
No	67(6.06%)	4.09 ± 2.37	4(3-6)	11,1	0.96^a^
Yes	1036(93.76%)	4.07 ± 2.61	4(2-6)	18,1	

**Storing pesticides**					
At home	60(5.43%)	3.6 ± 2.34	3(1-5)	11,1	0.34^a^
At the field	1034 (94.39)	4.12 ± 2.57	4(2-6)	18,1	

**Source of information**					
Agricultural engineer	390(35.29%)	3.78 ± 2.57	3(2-5)	13,1	0.01^a^
Others	713(64.52%)	4.23 ± 2.6	4(2-6)	18,1	

**Treating pesticides**					
No	550(49.77%)	4.19 ± 2.69	4(2-6)	14,1	0.14^a^
Yes	553(50.05%)	3.96 ± 2.5	4(2-6)	18,1	

**All pesticides are toxic**					
No	457(41.36%)	3.97 ± 2.36	4(2-6)	12,1	0.28^a^
Yes	646(58.46%)	4.15 ± 2.75	4(2-6)	18,1	

a ANOVA test

Among the participants in the study, the majority (715 individuals or 64.71%) were over 40 years old, and they reported a lower mean number of symptoms (4.36 ± 2.7) than those under 40. The level of education was found to have a significant effect on the number of reported symptoms (p = 0.01), with high school graduates reporting a lower mean number of symptoms (3.97 ± 2.58) in contrast to individuals who have not completed high school (4.45 ± 2.61). Farmers who worked in both greenhouse and open land crops reported a significantly higher mean number of symptoms (4.33 ± 2.65) than those who worked in only one type of crop (p = 0.01). The frequency and duration of pesticide spraying, not wearing a mask, wearing gloves, having a second job, not wearing eyeglasses, wearing special clothes, and the source of information were all significantly associated with the mean number of reported symptoms (p-values = 0.001a).

According to Table 2, the majority of farmers (59%) in the study reported experiencing health problems, with headaches being the most commonly reported symptom. Other frequently reported symptoms included itching, allergies, dizziness, shortness of breath, heavy sweating, blurred vision, skin problems, heavy tears, depression, tachycardia, and vomiting. However, 10% of farmers who used pesticides reported no adverse health effects. The study observed a range of symptom reports among participants. More than 18.28% of participants reported only one symptom, while 16.02%, 15.48%, 13.76%, 10.14%, 8.96%, 6.88%, 4.34%, 2.81%, and 1.72% reported two, three, four, five, six, seven, eight, nine, and ten symptoms respectively (Table 3). Moreover, approximately 22% of participants reported having 6-10 symptoms, and the highest number of symptoms reported by any participant was eighteen. Only a small percentage (less than 2%) reported having no symptoms and were thus excluded from the study.

**Table 2:** Frequency of neurological health symptoms among study participants

Neurological symptoms	Farmers n (%)
Headache	652 (59)
Itching	520 (47)
Allergy	416 (37)
Dizziness	403 (36)
Shortness of breath	375 (34)
Heavy sweeting	320 (29)
Blurring of vision	254 (23)
Skin problems	254 (23)
Heavy tears	23l (21)
Depression	263 (21)
Tachycardia	143 (13)
Vomiting	135 (12)
Heavy saliva	80 (7)
Frequent infection	58 (5)
Shivering	33 (3)
Nose bleeding	33 (3)
Weak memory	26 (2)

**Table 3:** The number of symptoms associated with pesticides poisoning

Number of symptoms	Farmers n (%)
1	202 (18.28%)
2	177 (16.02%)
3	171 (15.48%)
4	152 (13.76%)
5	112 (10.14%)
6	96 (8.69%)
7	76 (6.88%)
8	48 (4.34%)
9	31 (2.81%)
10	19 (1.72%)
11	9 (0.81%)
12	7 (0.63%)
13	3 (0.27%)
14	1 (0.09%)
17	1 (0.09%)
Total	1105 (100%)
n (Missing)	0 (0)
Mean ± Std Dev	4.07 ± 2.59
Median (Q1-Q3)	4 (2-6)
Min, Max	1, 17

Table 4 summarizes the findings of the study's univariate and multivariate models, which aimed to explore the association between the number of reported symptoms and different covariates. The results showed significant negative associations between the number of symptoms and the following variables: being above 40 years old (β = -0.49, 95%CI -0.89 to -0.08, p = 0.018), having a university degree (β = -0.41, 95%CI -0.8 to -0.02, p = 0.038), using pesticides less frequently per week (β = -0.82, 95%CI -1.15 to -0.49, p = 0.001), having another job (β = -0.49, 95%CI -0.82 to -0.16, p = 0.004), not wearing eyeglasses (β = -0.54, 95%CI -0.92 to -0.17, p = 0.005), and obtaining information from sources other than agriculture engineers or pesticide companies (β = -0.58, 95%CI -0.92 to -0.25, p = 0.001).

**Table 4:** The univariate and multivariate models of the association between a number of symptoms and other covariates

Variable	B (95%CI)	*p*-value	Adjusted B (95%CI)	*p*-value
**Age**				
>40	-0.43 (-0.76,-0.11)	0.009	-0.49 (-0.89,-0.08)	0.018
≤ 40	Ref		Ref	

**Gender**				
Male	0.11 (-0.42,0.64)	0.674	0.33 (-0.22,0.88)	0.234
Female	Ref		Ref	

**Material status**				
Unmarried	0.17 (-0.26,0.59)	0.439	-0.03 (-0.54,0.48)	0.896
Married	Ref		Ref	

**Smoking**				
Yes	-0.3 (-0.63,0.04)	0.088	-0.24 (-0.59,0.12)	0.191
No	Ref		Ref	

**Education (high school)**				
Yes	-0.48 (-0.85,-0.11)	0.012	-0.41 (-0.8,-0.02)	0.038
No	Ref		Ref	

**Years of working**				
< 10 years	0.09 (-0.31,0.48)	0.668	0.24 (-0.18,0.66)	0.255
>10 years	Ref		Ref	

**Types of crops**				
plastic houses	-0.78 (-1.35,-0.21)	0.007	-0.46 (-1.03,0.11)	0.115
Terrestrial crops	-0.39 (-0.71,-0.07)	0.016	-0.1 (-0.43,0.24)	0.58
Both	Ref		Ref	

**Frequency of spraying**				
1 /week	-0.99 (-1.31,-0.67)	<0.001	-0.82 (-1.15,-0.49)	<0.001
>1 /week	Ref		Ref	

**Spraying duration (min)**				
<30	0.61 (0.3,0.91)	<0.001	0.49 (0.18,0.8)	0.002
≥30	Ref		Ref	

**Wearing mask**				
No	-0.6 (-0.97,-0.24)	0.001	-0.11 (-0.57,0.34)	0.631
Yes	Ref		Ref	

**Washing hand**				
Yes	0.73 (-0.51,1.98)	0.248	0.65 (-0.65,1.96)	0.325
No	Ref		Ref	

**Eating or smoking while spraying**				
Yes				
No	0.04 (-0.59,0.67)	0.894	0.49 (-0.2,1.18)	0.161
	Ref		Ref	

**Wearing gloves**				
Yes	-0.61 (-0.98,-0.25)	0.001	-0.03 (-0.51,0.45)	0.896
No	Ref		Ref	

**Other work**				
Yes	-0.7 (-1.02,-0.38)	<0.001	-0.49 (-0.82,-0.16)	0.004
No	Ref		Ref	

**Wearing eyeglasses**				
No	-0.74 (-1.04,-0.43)	<0.001	-0.54 (-0.92,-0.17)	0.005
Yes	Ref		Ref	

**Washing vegetables**				
Yes	0.37 (-0.5,1.25)	0.404	0.12 (-0.8,1.05)	0.793
No	Ref		Ref	

**Special cloths**				
Yes	0.57 (0.25,0.9)	<0.001	0.35 (-0.04,0.74)	0.08
No	Ref		Ref	

**Reuse pesticide boxes**				
Yes	0.29 (-0.74,1.32)	0.578	-0.14 (-1.19,0.91)	0.797
No	Ref		Ref	

**Adhere to guidelines**				
Yes	-0.02 (-0.66,0.63)	0.961	-0.03 (-0.69,0.63)	0.926
No	Ref		Ref	

**Storing pesticides**				
In the field	0.54 (-0.2,1.28)	0.156	0.01 (-0.74,0.76)	0.981
At home	Ref		Ref	

**Source of information**				
Agricultural engineer	-0.45 (-0.77,-0.13)	0.006	-0.58 (-0.92,-0.25)	<0.001
Others	Ref		Ref	

**Do you treat all pesticides in the same way?**				
Yes	-0.23 (-0.54,0.08)	0.143	-0.28 (-0.59,0.03)	0.074
No	Ref		Ref	

**Are all pesticides toxic?**				
Yes	0.17 (-0.14,0.48)	0.279	0.35 (0.04,0.66)	0.028
No	Ref		Ref	

The model was adjusted to: Age, Gender, marital status, smoking, education, years of experience, types of crops, frequency of insecticides use, duration of spraying time, wearing mask, hands washing after work. Eating/smoking while spraying, wearing special cloths, other jobs, wearing eyeglasses, washing vegetables before eating, reuse of pesticide containers, adherence to guidelines, pesticides storing, source of information, ways of treating pesticides, toxicity of pesticides.

Additionally, the study found significant positive relationships between the number of symptoms and spraying period (β = 0.49, 95%CI 0.18 to 0.8, p = 0.002) and perception of pesticide toxicity (β = 0.35, 95%CI 0.04 to 0.66, p = 0.028). These findings suggest that longer spraying periods and the belief that pesticides are highly toxic are associated with an increased number of reported symptoms. On the other hand, certain protective measures, such as wearing eyeglasses and obtaining information from reliable sources, were found to be associated with a decreased number of reported symptoms.

## Discussion

The use of pesticides is widespread, especially in agriculture, to combat various harmful organisms, but it also carries serious health risks^17^. According to a study, the annual global death toll due to pesticide poisoning exceeds 300,000^18^. Younger farmers have been found to be more vulnerable to pesticide poisoning due to their limited work experience. Research has shown that there is a significant correlation between education level and the mean number of symptoms reported by farmers. This suggests that farmers with a higher level of education are more likely to be aware of the potential risks associated with pesticide use, which in turn leads to a lower number of reported symptoms. Previous studies have also highlighted that inadequate training and knowledge regarding the safe usage of pesticides can result in increased health hazards^19^. Previous studies have indicated that the variety of crops grown can have a substantial effect on the mean number of symptoms, potentially attributed to the utilization of a broader spectrum of pesticides^20^. Several epidemiological investigations have demonstrated that the intensity of symptoms is correlated with the frequency and length of exposure to pesticides^6^. Our research also revealed that the mean number of symptoms was impacted by the frequency of pesticide application and duration of exposure. To safeguard the well-being of farmers, it is crucial to utilize personal protective equipment (PPE) such as gloves, masks, coveralls, gowns, shoe covers, respirators, goggles, and face shields^21^. The information we gathered indicated that Palestinian farmers regularly employed PPE during various phases of pesticide management, and this had a notable impact on decreasing the average number of symptoms. A previous study in Palestine showed, the mean score for protective procedures was 9.8 (SD: 2.4; range: 3–14)^22^. In actuality, the implementation of PPE can decrease the likelihood of pesticide poisoning by nearly 44%^23^. It would be beneficial to provide more detailed insights into specific recommendations and strategies aimed at improving the utilization of PPE among farmers in Palestine. This could involve exploring tailored approaches, educational initiatives, or targeted interventions to address potential barriers and enhance the overall effectiveness of PPE implementation in the context of Palestinian agriculture. The involvement of agricultural engineers is crucial in resolving challenges related to the preservation and processing of agricultural commodities^24^. Our research uncovered that farmers who obtained guidance from agricultural engineers exhibited a lower average number of symptoms (3.78±2.57) in comparison to farmers who depended on untrained salespersons or other farmers (4.23±2.6). Agriculture engineers offer farmers expert advice, and agrochemical companies can supply the pesticides that they endorse.

Prior investigations have consistently demonstrated that people who handle pesticides are more likely to encounter a greater number of symptoms compared to those who do not. In this specific study, farmers self-reported experiencing a variety of symptoms, although these were not validated by medical experts. The symptoms reported by the participants were primarily associated with headaches, allergies, itching, dizziness, and shortness of breath, underscoring significant apprehensions related to exposure to pesticides. As previously noted in other studies, it is plausible that the utilization of different types of pesticides on various crops might have contributed to the occurrence of multiple symptoms^6^. In our study, farmers whreported being exposed to pesticides encountered a diverse range of symptoms, including but not limited to headache, itching, allergies, shortness of breath, dizziness, heavy sweating, blurred vision, skin issues, heavy tearing, and depression. These symptoms align with clinical evaluations since the frequency of self-reported symptoms is frequently associated with the intensity of harm caused by pesticide exposure^25^.

To establish the final adjusted model, a logistic regression analysis was performed using all significant analytical variables, as depicted in Table 4. The final model indicated that farmers under 40 years of age reported experiencing acute pesticide poisoning more frequently than those aged 40 and above (95% CI: -0.89, -0.08). This contradicts a previous study's findings that suggested acute pesticide exposure rises with increasing age^6^. Additionally, our study found that farmers with higher levels of education were more inclined to take additional precautions against pesticide poisoning (95% CI: -0.8, -0.02), while those with lower levels of education did not significantly mitigate their risk. However, in another investigation, the correlation between the level of education and the number of preventive measures taken was not evident^13^. Research has shown that improved education can enhance awareness of pesticides and how to use them correctly in low-income areas^6^.

Our study found that the frequency of spraying in each session (95% CI: -1.15, -0.49) and the duration of time spent spraying (95% CI: 0.18, 0.8) were associated with the toxicity of the pesticides used. It should be noted that symptoms of chronic exposure may take years to manifest, and the highest danger arises from pesticides that accumulate or decompose gradually in living tissues^6^. Our analysis indicated that eyewear had a protective effect (95% CI: -0.92, -0.17). However, it's worth noting that sunglasses may not offer sufficient protection as sprays or splashes can still enter around the edges.

The findings from our study, where the majority of farmers rely on information from peers and a limited proportion seek advice from agricultural engineers, underscore the prevailing gap in knowledge among farmers regarding pesticide hazards. This corroborates existing research emphasizing the need for enhanced education and awareness programs targeting farmers. The identified significant influence of the perception of pesticide toxicity on ensuring farmers' safety highlights the critical role of risk perception in promoting safer pesticide practices. This underscores the imperative for comprehensive educational initiatives that not only address the practical aspects of pesticide use but also foster a nuanced understanding of pesticide toxicity and associated risks. Recognizing the pivotal role of perception in shaping farmers' behavior is integral to developing targeted interventions that can effectively enhance safety practices and reduce health risks in the agricultural community.

## Conclusion

This study emphasizes the urgency of targeted interventions, including comprehensive education programs, peer-to-peer information exchange, and promotion of consistent PPE usage. Recommendations involve structured platforms for information sharing, subsidized or free PPE distribution, awareness campaigns, and collaboration with agricultural engineers. Advocacy for stronger regulations, enforcement of guidelines, and support for safer alternatives like bio-pesticides is essential. Investing in research and promoting sustainable practices, such as integrated pest management, can further contribute to a safer environment. These measures aim to reduce risks associated with pesticide exposure and enhance longterm agricultural resilience.

## References

[1] Oerke E (2006). Crop losses to pests. The Journal of Agricultural Science.

[2] al-Saleh IA (1994). Pesticides: a review article. J Environ Pathol Toxicol Oncol.

[3] Davies JE, Freed VH, Whittemore FW (1990). An agromedical approach to pesticide management: Some health and environmental considerations. An agromedical approach to pesticide management: Some health and environmental considerations: US Agency for International Development (AID); Consortium for International ….

[4] Pingali PL, Marquez CB, Palis FG, Rola AC, Pingali PL, Roger PA (1995). The Impact of Pesticides on Farmer Health: A Medical and Economic Analysis in the Philippines. Impact of Pesticides on Farmer Health and the Rice Environment.

[5] Prashar A, Ramesh M (2018). Assessment of pattern and outcomes of pesticides poisoning in a tertiary care hospital. Tropical Medicine & International Health.

[6] Damalas CA, Koutroubas SD (2016). Farmers' Exposure to Pesticides: Toxicity Types and Ways of Prevention. Toxics.

[7] Thundiyil JG, Stober J, Besbelli N, Pronczuk J (2008). Acute pesticide poisoning: a proposed classification tool. Bulletin of the World Health Organization.

[8] AgriService PM (2010). Industry Overview: 2009 Market.

[9] Gianessi L (2009). The value of insecticides in US crop production. Croplife Foundation.

[10] Raimi MO (2021). Self-reported Symptoms on Farmers Health and Commonly Used Pesticides Related to Exposure in Kura, Kano State, Nigeria. Morufu Olalekan Raimi. “Self-reported Symptoms on Farmers Health and Commonly Used Pesticides Related to Exposure in Kura, Kano State, Nigeria”. Annals of Community Medicine & Public Health.

[11] Fan L, Niu H, Yang X, Qin W, Bento CP, Ritsema CJ (2015). Factors affecting farmers' behaviour in pesticide use: Insights from a field study in northern China. Science of the Total Environment.

[12] Abdollahzadeh G, Sharifzadeh MS, Damalas CA (2016). Motivations for adopting biological control among Iranian rice farmers. Crop Protection.

[13] Hashemi SM, Rostami R, Hashemi MK, Damalas CA (2012). Pesticide use and risk perceptions among farmers in southwest Iran. Human and Ecological Risk Assessment: An International Journal.

[14] Riccò M, Vezzosi L, Gualerzi G (2018). Health and safety of pesticide applicators in a high income agricultural setting: a knowledge, attitude, practice, and toxicity study from North-Eastern Italy. Journal of Preventive Medicine and Hygiene.

[15] Zyoud SeH, Sawalha AF, Sweileh WM, Awang R, Al-Khalil SI, Al-Jabi SW (2010). Knowledge and practices of pesticide use among farm workers in the West Bank, Palestine: safety implications. Environmental Health and Preventive Medicine.

[16] Al-Rujoub R, Khatib I, Al-Shami Nm, Salahat J (2019). Occupational safety and health practices among farmers in Wadi Al Fara'Area, Palestine.

[17] Ngowi A, Mrema E, Kishinhi S (2016). Pesticide Health and Safety Challenges Facing Informal Sector Workers: A Case of Small-scale Agricultural Workers in Tanzania. NEW SOLUTIONS. A Journal of Environmental and Occupational Health Policy.

[18] Aidoo AK, Arthur S, Bolfrey–Arku G, Mochiah MB (2019). Pesticides abuse and health implications in Ghana: A review. International Journal of Environment, Agriculture and Biotechnology.

[19] Groot MJ, Van't Hooft KE (2016). The Hidden Effects of Dairy Farming on Public and Environmental Health in the Netherlands, India, Ethiopia, and Uganda, Considering the Use of Antibiotics and Other Agro-chemicals. Frontiers in Public Health.

[20] Zhang X, Zhao W, Jing R, Wheeler K, Smith GA, Stallones L (2011). Work-related pesticide poisoning among farmers in two villages of Southern China: a cross-sectional survey. BMC Public Health.

[21] Chughtai AA, Chen X, Macintyre CR (2018). Risk of self-contamination during doffing of personal protective equipment. American Journal of Infection Control.

[22] Zyoud SH, Sawalha AF, Sweileh WM, Awang R, Al-Khalil SI, Al-Jabi SW (2010). Knowledge and practices of pesticide use among farm workers in the West Bank, Palestine: safety implications. Environmental Health and Preventive Medicine.

[23] Dasgupta S, Meisner C, Wheeler D, Xuyen K, Thi Lam N (2007). Pesticide poisoning of farm workers–implications of blood test results from Vietnam. International Journal of Hygiene and Environmental Health.

[24] Atay CE, Ayebare P (2017). Determination of buffer-zones using agricultural information system. TEM Journal.

[25] Tschudi-Madsen H, Kjeldsberg M, Natvig B, Ihlebaek C, Dalen I, Straand J (2013). Multiple symptoms and medically unexplained symptoms — Closely related concepts in general practitioners' evaluations. A linked doctor–patient study. Journal of Psychosomatic Research.

[26] Mubushar M, Aldosari FO, Baig MB, Alotaibi BM, Khan AQ (2019). Assessment of farmers on their knowledge regarding pesticide usage and biosafety. Saudi Journal of Biological Sciences.

